# Non‐invasive coronary CT angiography‐derived fractional flow reserve: A benchmark study comparing the diagnostic performance of four different computational methodologies

**DOI:** 10.1002/cnm.3235

**Published:** 2019-08-16

**Authors:** Jason Matthew Carson, Sanjay Pant, Carl Roobottom, Robin Alcock, Pablo Javier Blanco, Carlos Alberto Bulant, Yuri Vassilevski, Sergey Simakov, Timur Gamilov, Roman Pryamonosov, Fuyou Liang, Xinyang Ge, Yue Liu, Perumal Nithiarasu

**Affiliations:** ^1^ Zienkiewicz Centre for Computational Engineering, College of Engineering Swansea University Swansea UK; ^2^ Data Science Building, Swansea University Medical School Swansea University Swansea UK; ^3^ Derriford Hospital and Peninsula Medical School Plymouth Hospitals NHS Trust Plymouth UK; ^4^ Department of Mathematical and Computational Methods National Laboratory for Scientific Computing, LNCC/MCTIC Petrópolis Brazil; ^5^ National Scientific and Technical Research Council (CONICET) Buenos Aires Argentina; ^6^ Marchuk Institute of Numerical Mathematics Russian Academy of Sciences Moscow Russia; ^7^ Laboratory of Human Physiology Moscow Institute of Physics and Technology Moscow Russia; ^8^ Institute of Personalized Medicine, Laboratory of Mathematical Modelling in Medicine Sechenov University Moscow Russia; ^9^ School of Naval Architecture, Ocean and Civil Engineering Shanghai Jiao Tong University Shanghai China

**Keywords:** benchmark, fractional flow reserve, haemodynamic models

## Abstract

Non‐invasive coronary computed tomography (CT) angiography‐derived fractional flow reserve (cFFR) is an emergent approach to determine the functional relevance of obstructive coronary lesions. Its feasibility and diagnostic performance has been reported in several studies. It is unclear if differences in sensitivity and specificity between these studies are due to study design, population, or "computational methodology." We evaluate the diagnostic performance of four different computational workflows for the prediction of cFFR using a limited data set of 10 patients, three based on reduced‐order modelling and one based on a 3D rigid‐wall model. The results for three of these methodologies yield similar accuracy of 6.5% to 10.5% mean absolute difference between computed and measured FFR. The main aspects of modelling which affected cFFR estimation were choice of inlet and outlet boundary conditions and estimation of flow distribution in the coronary network.

One of the reduced‐order models showed the lowest overall deviation from the clinical FFR measurements, indicating that reduced‐order models are capable of a similar level of accuracy to a 3D model. In addition, this reduced‐order model did not include a lumped pressure‐drop model for a stenosis, which implies that the additional effort of isolating a stenosis and inserting a pressure‐drop element in the spatial mesh may not be required for FFR estimation.

The present benchmark study is the first of this kind, in which we attempt to homogenize the data required to compute FFR using mathematical models. The clinical data utilised in the cFFR workflows are made publicly available online.

## Graphical abstract

In this work four different computational FFR workflows are compared with invasive clinical measurements, which includes a 3D methodology, and three reduced‐order methodologies. The results indicate that there is a not‐so‐evident sensitivity with respect to the methodology as three of the four methodologies produces similar levels of accuracy. The clinical data utilised in this work has been made freely available online.

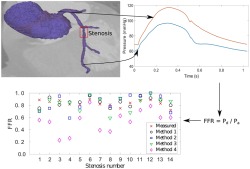

## 1 INTRODUCTION

Coronary computed tomography angiography (CCTA) is a well‐established imaging technique for the diagnosis and risk stratification of patients with coronary artery disease (CAD).^1^, ^2^ However, CCTA fails to predict the physiological or functional significance of coronary artery stenoses.^3^ Invasive coronary catheter angiography‐based measurements of fractional flow reserve (FFR) is the current gold standard of care for the functional assessment of coronary artery obstructive lesions.^4^, ^5^ When compared with quantitative coronary angiography (QCA) alone, an FFR‐guided strategy has been shown to reduce unnecessary stenting, improve overall health outcome, and to be cost‐saving.^6^, ^7^, ^8^, ^9^ With a significant predicted increase in the prevalence of coronary heart disease worldwide,^10^, ^11^, ^12^ the already substantial economic burden of this disease will likely increase. Therefore, there is a compelling need to further reduce diagnosis and treatment costs while maintaining or improving the standard of care.

Recently, the use of CCTA‐based non‐invasive computation of FFR (referred to simply as cFFR) has been proposed as a virtual surrogate to estimate the functional impact of coronary stenoses. The relevance of such an approach is that, through mathematical modelling and computer simulation, it makes possible the integration of anatomical and physiological information. Most approaches which rely on computational modelling employ semi‐automated algorithms to segment the patient‐specific coronary geometry, on top of which blood flow simulations are conducted. Additional information based on the patients' physiological conditions are utilised in the computational model to estimate boundary conditions. These boundary conditions are usually expressed in terms of prescribed flow rate and pressure at the proximal and distal artificial interfaces created when isolating the vessels in the coronary network. There exist several cFFR approaches which include the use of computationally expensive three‐dimensional models,^13^, ^14^, ^15^, ^16^, ^17^, ^18^, ^19^, ^20^ or the use of dimensionally reduced‐order models.^21^, ^22^, ^23^, ^24^, ^25^, ^26^, ^27^, ^28^, ^29^, ^30^ Critical components of all methodologies include (a) the accuracy of CCTA image segmentation and (b) the choice/estimation of boundary conditions.

Moreover, in order to accurately estimate FFR, the computational model must be able to replicate the haemodynamic environment in the coronary system during hyperaemic conditions. Normally, this condition of increased flow is modelled by assuming a proportional increase in the flow rate with respect to the flow rate corresponding to the resting state. Hence, the total coronary peripheral resistance during maximum hyperaemia is postulated to be a fraction of its value at rest.^31^ Clearly, the estimated distribution of this peripheral resistance through the coronary branches is critical as it determines the distribution of coronary flow. This is often performed using allometric laws relating flow rate to vessel diameter^18^, ^23^, ^32^ as well as from the downstream myocardial mass.^33^, ^34^

The particularity of this research requires gathering and utilising patient data (CT scans, clinical data, and FFR values), which is not always possible. Currently, there exists no openly available resource to allow comparison of new methodologies with currently published methodologies for cFFR.

The aim of the present benchmark study is primarily to make available the necessary clinical data to estimate FFR through the use of mathematical models. We also report the diagnostic performance of four different methodologies to estimate cFFR. Each methodological approach is described in detail with full supporting information. The cFFR and the invasively measured FFR are made available along with the information necessary to conduct computer simulations for each patient included in this study. We strongly believe that this study will result in a valuable initiative towards the benchmarking and standardization of alternative methodologies in the field of non‐invasive estimation of FFR. All methodologies were tested using a cohort of 10 patients, with a total of 14 stenotic lesions. The tests were performed with access to anonymized patient data, which included CCTA images, patient blood pressure (aortic or external cuff) at rest, and basic clinical information. Concerning the values of the FFR, the study was fully blinded. The invasively measured FFR values were disclosed once all simulations were completed and shared. The methodologies were tested on CCTA data with varied quality, including challenging cases with significant calcification and/or stents.

## 2 MATERIALS AND METHODS

### 2.1 Study characteristics

A cohort of 10 patients was chosen for this retrospective study. Patient information was anonymized and de‐identified by clinicians prior to being sent to research groups for cFFR analysis. The CCTA was performed in each patient before catheterisation (coronary angiogram and invasive FFR). Patients who had undergone previous coronary surgical interventions were only included if the previous intervention occurred within vessels, which were of no interest for this study. Patient cases were carefully chosen based on the quality of the CCTA scans to give a spread of both good quality images, and images which included calcified regions and some motion artefacts, making the segmentation of coronary vessels more challenging. An overview of the patient CCTA data is given as follows:

- Patient 1 ‐ small motion artefacts were present in the image at the base of the heart, segmentation performed without difficulty.
- Patient 2 ‐ calcification in the left anterior descending artery (LAD) which may have a minor effect on segmentation.
- Patient 3 ‐ motion artefacts in the image with minor calcification of the right coronary artery (RCA), which may have a minor effect on segmentation.
- Patient 4 ‐ stent present in the obtuse marginal artery (OM), with significant calcification in the LAD which may affect segmentation accuracy.
- Patient 5 ‐ calcification in the LAD which may have a minor effect on segmentation.
- Patient 6 ‐ calcification in the diagonal artery (DA) close to bifurcation with the LAD. Segmentation of distal LAD and DA difficult to perform and may be affected by the calcified region.
- Patient 7 ‐ calcification in the left circumflex artery (LCx) and LAD causing minor difficulties in segmentation.
- Patient 8 ‐ minor calcification in LAD which is not likely to effect segmentation accuracy.
- Patient 9 ‐ significant calcification in left coronary artery (LCA), LCx, and LAD which may affect accuracy of the segmentation.
- Patient 10 ‐ significant calcification of the LAD, which may affect accuracy of the segmentation.

Complementarily to CCTA images, additional patient information were necessary to compute cFFR. The information included patient age, blood pressure at rest (aortic or brachial artery), heart rate, height, and weight. An overview of the patient information is given in Table 1. Artery and lesion characteristics are presented in Table 2. All four methodologies were blinded to the invasive measure of FFR. After all cFFR simulations were completed, the simulated results were reported to a clinician before each group received the actual FFR measurements for analysis.

**Table 1:** Patient description

Patient no.	Age	HR	Blood Pressure	Height	Weight	BMI	Gender	Circulation
	(Y)	(BPM)	(mmHg)	(cm)	(kg)			Dominance
1	80	67	174 / 76 / 111	168	88	32.0	F	L
2	64	80	104 / 65 / 83	182	124	36.0	M	R
3	57	72	187 / 83 / 125	173	85	25.4	F	R
4	68	88	130 / 66 / 94	NA	78.4	25.0	M	L
5	52	73	138 / 74 / 99	NA	NA	33.0	F	L
6	53	48	142 / 70 / 99	NA	NA	25.0	F	R
7	56	48	140 / 73 / 98	183	54	31.0	M	R
8	50	85	133 / 86 / 110	172.7	92.1	28.0	M	R
9	66	75	111 / 76 / NA	173	88	29.4	M	R
10	67	58	130 / 60 / 108	170	68.8	23.8	M	R

*Note*. Missing values are indicated by NA. Blood pressure is expressed as systolic/diastolic/mean and denotes the aortic pressure at rest measured invasively (for all patients except for patient 9, which is the cuff pressure at the brachial artery, also at rest). HR is the resting heart rate in beats‐per‐minute at the time of CCTA. Circulation dominance is defined from the arteries visible in the CCTA image as L or R

Abbreviations: BMI, body mass index; CCTA, coronary computed tomography angiography; HR, heart rate; L, left; R, right.

**Table 2:** Distribution of interrogated arteries and lesions among the patient sample

Interrogated Artery (*n* = 14)	n (%)
LAD	10 (72)
DA	1 (7)
LCx	2 (14)
RCA	1 (7)
**Lesion characteristic (*n* = 14)**	**mean**±**SD (Q1‐Q3)**
FFR	0.84±0.05 (0.82‐0.88)
Percentage diameter stenosis (%)	46.00±10.49 (40‐55)

*Note*. Percentage diameter stenosis is measured by physicians directly from the CCTA image.

Abbreviations: CCTA, coronary computed tomography angiography; DA, diagonal artery; FFR, fractional flow reserve; LAD, left anterior descending artery; LCx, left circumflex artery; RCA, right coronary artery.

Since this study aims to show differences in the estimation of FFR from different cFFR computational methodologies, the shared data and methods were kept to a minimum, which implies that each research group:

1. performed the image segmentations and built the computational meshes;
2. chose the mathematical model and established the criteria to define the boundary conditions;
3. estimated the main simulation parameters, ie, hyperemic coronary blood flow and flow distribution at per‐artery level; and
4. defined the location of cFFR measurements based on the sketches reported by the physicians.

It is important to highlight that each of these points presented above is a source of discrepancy among all computational methodologies, and will be discussed in Section 3.

### 2.2 CCTA acquisition and invasive FFR

Standard CCTA protocols were performed. Metoprolol (beta blocker) was administered intravenously for some patients to moderate heart rate and improve image quality during scanning. The tube potential used in the scans ranged between 100 and 120 kV with prospective gating and zero padding. Prospective gating uses an electrocardiograph as a trigger to scan at a particular point in the cardiac cycle. Several of the scans were performed in high definition/resolution mode. The average in‐plane pixel spacing was 0.458±0.051*mm*, and the slice‐spacing was 0.625*mm*. All CCTA images were provided in (anonymized) DICOM format.

During invasive FFR measurements, each patient received an intravenous infusion of adenosine to trigger a hyperaemic condition. The FFR measurements in catheter angiography were performed during maximum hyperaemic conditions. FFR was calculated using the mean aortic pressure (see Table 1) and mean pressure distal to the stenosis as measured by the pressure sensitive catheter. The diagnostic cut‐off threshold for positive FFR was chosen to be 0.8.

### 2.3 Non‐invasive cFFR

A critical component for the accurate prediction of cFFR is the estimation of boundary conditions such as stroke volume / cardiac output and regional distribution of vascular resistance in the coronary network. The estimation of these parameters will be described for each methodology in Section 2.4. The CCTA image data received by the research groups corresponded to a single phase (75% diastole). This enables the segmentation procedure to be performed (assuming sufficient image quality), but it is not sufficient to estimate heart function and, thus, parameters such as stroke volume cannot be retrieved from the images.^35^ Through the use of basic clinical data, several cardiac parameters can be estimated. Cardiac output/stroke volume can be estimated from height and weight measurements (or body mass index [BMI]),^36^, ^37^ from cardiac index,^38^ from body surface area,^39^ from age,^40^, ^41^ or from a combination of heart rate and blood pressure.^42^ However, these measures are susceptible to significant errors, in particular with respect to estimation of cardiac index for individuals with a higher BMI.^43^ In turn, the physiological effect of hyperaemia can be estimated from cardiac output at rest^44^ as well as the distribution of hyperaemic flow in the coronary arteries.^45^

The cFFR value was computed as cFFR $=\frac{P_{d}}{P_{a}}$ for all cases, where *P*_*a*_ is either the mean aortic pressure, or a mean pressure at a point proximal to the stenosis (depending on the methodology), and *P*_*d*_ is the mean pressure distal to the stenosis. The location in the interrogated artery for the *P*_*d*_ measurement is independently defined by each research group following the same set of diagrams provided by the physicians that conducted the invasive study. Such diagrams are part of the public data associated to this study.

### 2.4 Methodological approaches to estimate cFFR

This section outlines the four methodologies used to compute cFFR. We focus on highlighting common and particular aspects of each methodology, from the construction of the geometrical model using CCTA data (3D, reduced‐order models or a combination of both), the prescription of a physiological model, ie, the boundary conditions, to the physical model and the computational strategy used to solve for the coronary blood flow and pressure.

In what follows, the methodologies are presented in no particular order, and

- Methodology 1: Developed at Zienkiewicz Centre for Computational Engineering, College of Engineering, Swansea, UK.
- Methodology 2: Developed at HeMoLab group (http://hemolab.lncc.br) from the National Laboratory for Scientific Computing, Petrópolis, Brazil.
- Methodology 3: Shanghai Jiao Tong University, China.
- Methodology 4: Marchuk Institute of Numerical Mathematics, Russian Academy of Sciences, Moscow, Russia.

#### 2.4.1 Methodology 1

The first approach uses a 1D‐0D model to represent the patient‐specific coronary model. Segmentation of the CCTA data, computation of centrelines, and extraction of vessel geometry were performed using the commercial image segmentation software VMTKLab, (Orobix, Italy). The governing equations for the 1D model are given by

(1) $$\left\{\begin{array}{l} C_{A}\frac{\partial P}{\partial t}+\frac{\partial Q}{\partial x}=0,\\ \frac{\rho }{A}\frac{\partial Q}{\partial t}+\frac{\rho }{A}\frac{\partial \left(\frac{Q^{2}}{A}\right)}{\partial x}+\frac{\partial P}{\partial x}=\frac{-22\mu \pi Q}{A^{2}}, \end{array}\right.$$

where *C*_*A*_ is the compliance, *P* is the hydrostatic pressure, *Q* is the volumetric flow rate, *A* is the cross‐sectional area *ρ*=1.06 g/cm^3^ is the density of blood, and *μ*=0.04 P is the dynamic viscosity, *t* and *x* are the temporal and spatial coordinates, respectively. The equations are solved implicitly using a sub‐domain collocation scheme referred to as the enhanced trapezoidal rule method.^46^, ^47^ In this framework, no additional pressure drop model for the stenosis is introduced. The viscous friction term on the right side of the momentum equation is responsible for predicting the pressure drop due to the vessel narrowing. A fine spatial mesh of 0.1 mm is required to accurately account for sudden changes in geometry. The system is closed with the non‐linear visco‐elastic constitutive law^48^

(2) $$P-P_{ext}-P_{0}=\frac{2\rho c_{0}^{2}}{b}\left({\left(\frac{A}{A_{0}}\right)}^{b/2}-1\right)+\frac{\Gamma }{A_{0}\sqrt{A_{0}}}\frac{\partial A}{\partial t},\;b=\frac{2\rho c_{0}^{2}}{P_{0}-P_{collapse}},$$

where *P*_*ext*_ is the external pressure, *P*_0_ is a reference pressure, *P*_*collapse*_ is a collapsing pressure, *A*_0_ is the cross‐sectional area at the reference pressure, and *c*_0_ is the reference wave speed of the vessel calculated as

(3) $$c_{0}=\sqrt{\frac{2}{3\rho }\left(k_{1}\exp(k_{2}r_{0})+k_{3}\right)},$$

with $k_{1}=2.00\times 10^{7}{\;g}^{2}\text{/cm/s}$, $k_{2}=-22.53{\;\text{cm}}^{-1}$, $k_{3}=8.65\times 10^{5}g^{2}\text{/cm/s}$, and *r*_0_ is the reference radius of the vessel. The viscous wall coefficient is given by

(4) $$\Gamma =\frac{100}{2r_{0}}+100.$$

An initial simulation of a closed‐loop system^48^ is implemented with an adaptive parameter estimation technique^47^, ^49^ to achieve realistic cardiac output during hyperaemia^44^ of 7.6 L/min for all patients. The resistance in the coronary network was initially distributed by assuming the total coronary blood flow to be approximately 4.5 % of the cardiac output. Coronary blood flow during hyperaemia was then found by reducing coronary resistances by 78 %, which increases coronary flow rates by approximately 3.5 times of resting values^44^ and are in the observed range for hyperaemic conditions.^45^ The main purpose of this closed‐loop system is to determine the inflow waveforms of the left/right coronary arteries and left/right ventricular pressures which are used as boundary conditions in the patient specific coronary model. The left/right ventricular pressures, obtained via the lumped heart model in the closed‐loop system, are then used as compression pressures on the coronary vascular bed model. Using the closed‐loop system in conjunction with patient pressures and heart rate, the estimated flow rates into the coronary arteries will differ slightly between patients; in the right coronary artery, the flow is estimated to be 207.83 ml/min (only required for patient 3), while the left coronary artery has an estimated flow rate between 325.11 and 453.75 ml/min. Due to the level of uncertainty of patient measurements, and unmeasured data (such as flow rates), coronary dominance was not considered in this modelling approach.

**Figure 1:**
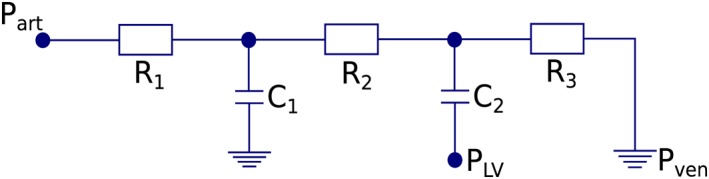
A lumped‐parameter model connected to the outlets of the patient specific coronary network to represent the micro‐circulation. *P*_*art*_ connects to the 1D domain, *R*_1_ is the characteristic impedance, *R*_2_ is the resistance of the micro‐circulation at the arterial side, *R*_3_ is the micro‐circulatory resistance at the venous side, *C*_1_ is the micro‐circulatory arterial compliance, *C*_2_ is the intra‐myocardial compliance, *P*_*LV*_ is a scaled pressure from the left ventricle (or right ventricle for the right coronary artery [RCA]), and *P*_*ven*_ represents the pressure in the venous system which is set to 5mmHg

The total resistance of each coronary branch *R*_*cor*,*i*_ is then calculated from the relation

(5) $$R_{cor,i}=\frac{MAP}{Q_{cor,i}},$$

where *MAP* is the mean arterial pressure from patient measurements and *Q*_*cor*,*i*_ is the mean defined inflow rate for each coronary branch as determined by the closed loop system. The distribution of resistance throughout each branch is determined using a variant of Murray's power law, with a power of 2.27 as in van der Giessen et al,^50^ with vascular bed compliance distributed in a similar way.^23^ The coronary vascular bed model is shown in Figure 1, which includes an external pressure acting from the heart ventricles.

#### 2.4.2 Methodology 2

The computational workflow consists of five main stages as follows:

1. Input of medical data: This stage includes the CCTA image and patient clinical data.
2. Image processing: Image segmentation of CCTA images is achieved using the methodology detailed in Bulant et al,^51^ where segmentation is performed using a level‐set method, initialized using a colliding front algorithm.^52^ This results in a triangulated raw surface (coarse triangular mesh) of the coronary tree.
3. Mesh processing and arterial network modeling: The coarse mesh is further processed to obtain the computational mesh suitable for 3D CFD simulations. Surface mesh processing steps include smoothing, incorporation of tube extensions at inlet/outlets, and adaptive refinement (function of vessel cross‐sectional radius). Finally, a tetrahedral volume mesh is constructed for the CFD simulations. An expanded explanation of the mesh processing pipelines used to obtained the CFD meshes can be found in Bulant.^53^ The vessel cross‐sectional radius is given by the arterial tree centreline, which is obtained following Antiga et al.^54^ The centreline is then processed to account for a bifurcation mask that defines the arterial ostium of each artery, and the anatomical name of each artery is assigned as labels, see Bulant et al^51^ for details.
4. Parameters setup and simulation: Patient‐specific parameters are estimated and used to define the boundary conditions for the computational simulations. Blood flow is modeled using the Navier‐Stokes equations for rigid domains, ie, arterial compliance is neglected. A Neumann boundary condition is considered at the inlet of the network, and resistance boundary conditions are considered at the outlets. The methodology used to enforce a given coronary blood flow using such resistance‐type terminals is explained elsewhere.^53^, ^55^ Steady‐state simulations are performed, maintaining all parameters constant.
  The blood density and viscosity are the same for all patients, *ρ*=1.05 g/cm^3^ and *μ*=0.04 P, respectively. The hyperaemic mean pressure at the root of the coronary arterial tree (*P*_*p*_) is estimated for each patient as *P*_*p*_=MP+Δ. Where MP is the mean aortic pressure (in mmHg) and Δ=−3.8 mmHg is the effect of intra‐coronary administration of adenosine, as reported in Bulant.^53^ The MP is given for all patients except Patient 9, see Table 1. In that case, the MP is estimated from diastolic (DP) and systolic (SP) pressures as (2DP+SP)/3.
  The resting coronary blood flow (RCBF) is estimated from patient clinical data. Specifically, it is well accepted that the RCBF is 4.5% of the cardiac output (CO).^56^ The CO is defined as the heart rate (HR) times the stroke volume (SV). Moreover, the SV is estimated from patient weight, age, heart rate, systolic and diastolic pressures.^57^ For patients {5,6}, the weight was estimated as the sample mean, and for patients {4,5,6} the height was estimated using the weight and the BMI, see Table 1.
  For the patient sample, the estimated RCBF is 5.63±1.97 ml/s, which is in the physiological range (4.5±1.37 ml/s) reported by.^58^ Coronary flow reserve (CFR) is defined as the ratio between hyperaemic and resting blood flow, and in nonischemic human coronary arteries, CFR mean value is ∼2.6^59^. By assuming CFR=2.6, the hyperaemic flow is CBF=CFR×RCBF=2.6×RCBF.
  Terminal resistances for a given coronary network are estimated using a variant of Murray's power law^60^ (exponent set to 2.66 motivated by allometric laws relating flow to volume of tissue^61^). Moreover, the radius of the arterial vessel employed in the power law is that corresponding to the ostium of the artery. Also, the distribution of CBF at the inlet of the major coronary branches (LAD, LCx, and RCA) follows the data reported in Table 3. Details of the algorithm used to estimate terminal resistances based on inflow, the hyperaemic mean pressure at the coronary root (*P*_*p*_), ostium radius, and a reference venous pressure (*P*_ref_=10 mmHg) are described in Bulant.^53^ For the study sample, the average resting flows at the inlet of the LAD, LCx, and RCA arteries are 3.38±1.18 ml/s, 1.40±0.85 ml/s, and 0.85±0.33 ml/s.
  **Table 3:** Percentage of the CBF at the inlet of each major artery as a function of circulation dominance

Circulation Dominance	LAD	LCx	RCA
Right	60	22	18
Left	60	30	10

Abbreviations: CBF, coronary blood flow; LAD, left anterior descending artery; LCx, left circumflex artery; RCA, right coronary artery.
5. Postprocessing and data analysis: After the execution of the simulation, the velocity (**v**) and pressure (*p*) fields are available at each node in the computational mesh. Estimation of cFFR field needs a proximal pressure *P*_*a*_, which is calculated as the spatial average at the inlet region of approximately 2 mm length. Such region is manually defined using points of the centreline, which are used to clip the tetrahedral mesh. Then, a new field containing the FFR at each computational node is calculated as
  (6) $$\text{FFR}(x)=\frac{p(x)}{P_{a}},$$
  Thus, the cFFR value is estimated as the average of the FFR(**x**) at a distal region of approximately 2 mm length, manually defined. The definition of such region was guided by sketches of the arterial tree drawn by physicians. It is worth noting that other haemodynamic variables such as the flow per terminal and the wall shear stress field can be extracted for data analysis.

All image processing stages, as well as meshing and centreline processing, are performed using vmtk,^62^ ImageLab,^63^ and HeMoLab^64^ softwares.

#### 2.4.3 Methodology 3

**Figure 2:**
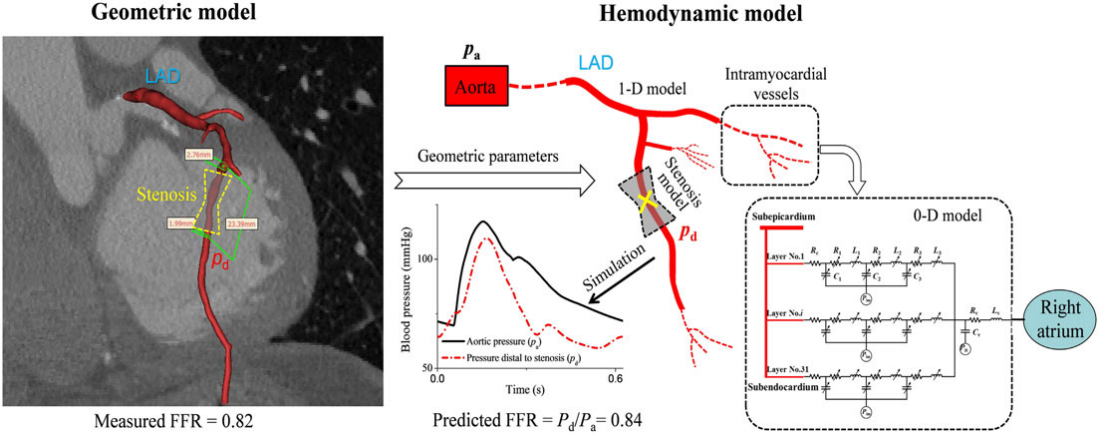
Schematic description of the methodology of methodology 3

The third methodology mainly involved image‐based reconstruction of the geometric model, extraction of geometric data, development of hemodynamic model, and prediction of FFR (see Figure 2 for a schematic description of the procedure). 3D geometric models of large epicardial coronary arteries were reconstructed from CCTA images using a commercial image‐segmentation software, Mimics 15.0 (Materialise, Belgium). The geometric models were subsequently compartmentalized into arterial segments according to the anatomical distribution of bifurcation and stenosis to facilitate the extraction of geometric data. Extracted geometric data for each arterial segment included the length, nominal diameters of the proximal and distal ends, or stenosis rate. These data were incorporated into a 0‐1D multi‐scale hemodynamic model of the coronary circulation that will be used to simulate coronary blood flow and predict FFR. Details on the development of the model have been provided elsewhere.^65^ In brief, the network of epicardial coronary arteries was represented by a 1D model, with its proximal ends being coupled to a 0‐1D model of the global cardiovascular system^66^ and distal ends connected to 0D (ie, lumped parameter) models of intramyocardial vessels. For the modeling of a stenosis where blood flow patterns are highly complex due to the presence of abrupt changes in lumen area, an empirical formula was employed to relate trans‐stenosis pressure drop to flow rate and major geometric parameters (eg, stenosis rate and stenosis length) of the stenosis.^67^

With regard to the assignment of model parameters not derivable directly from medical images, parameter optimizations were implemented to fit model‐simulated systemic arterial blood pressures, cardiac output, and blood flow rates in the major coronary arteries (ie, the LAD, the LCx, and the RCA) under both resting and hyperaemic conditions to population‐averaged data reported in the literature.^44^, ^45^ In addition, the resistances of intra‐myocardial vessels (represented by 0‐D models) distal to all the branch arteries originating from each major coronary artery were assumed to be identical for the purpose of simplicity. As a consequence, the haemodynamic model was only partly personalized (by incorporating patient‐specific geometric data of large coronary arteries and stenoses), with most model parameters being kept at a population‐averaged state.

The procedure of FFR measurement was simulated by applying the general hyperaemic conditions to the model through adjusting heart rate (from 66‐96 beats/min) and model parameters that represent cardiac contractility, systemic vascular resistance, and coronary microvascular resistance. Major hemodynamic changes from the resting condition to a hyperaemic physiological state included arterial systolic/diastolic pressure from 113/77 to 116/69 mmHg, cardiac output from 5.13 to 7.5 L/min, and blood flow rates in major coronary arteries. The flow rates LAD increased from 75.38 to 258.21 ml/min, in the LCx from 54.20 to 162.02 ml/min, and from 67.47 to 215.17 ml/min in the RCA. The calculation of virtual FFR followed the standard method where FFR was taken to be the ratio of poststenosis mean blood pressure to mean blood pressure in the aorta.

#### 2.4.4 Methodology 4

Patient‐specific geometries were obtained from the CT images by the method of automatic CT scans processing,^68^ which consists of four stages: aorta segmentation, computation of Frangi vesselness, ostia points detection, and coronary vessel segmentation, skeletonization of segmented vessels, and graph construction. Frangi vesselness filter is applicable for discontinuous and moving structures and thus is appealing for segmentation of coronary vessels with lesions.

The 1D model^30^, ^69^ is based on equations of mass and momentum conservation

(7) $$\frac{\partial A_{k}}{\partial t}+\frac{\partial \left(A_{k}u_{k}\right)}{\partial x}=0,$$

(8) $$\frac{\partial u_{k}}{\partial t}+\frac{\partial \left(u_{k}^{2}/2+p_{k}/\rho \right)}{\partial x}=-\frac{8\pi \mu u_{k}}{A_{k}},$$

where *k* is the index of the vessel, *t* is the time, *x* is the distance along the vessel, *ρ* is the blood density (constant), *A*_*k*_(*t*,*x*) is the vessel cross‐section area, *p*_*k*_ is the blood pressure, *u*_*k*_(*t*,*x*) is the linear velocity averaged over the cross‐section, and *μ* is the blood viscosity. Constitutive law for the material of the vessel wall is^70^

(9) $$p_{k}\left(A_{k}\right)-p_{*k}=\rho_{w}c_{k}^{2}f\left(A_{k}\right),$$

where *ρ*_*w*_ is vessel wall density (constant),

(10) $$f\left(A_{k}\right)=\left\{\begin{array}{ll} \exp\left(A_{k}/{A_{0}}_{k}-1\right)-1, & A_{k}/{A_{0}}_{k}>1\\ \ln A_{k}/{A_{0}}_{k}, & A_{k}/{A_{0}}_{k}⩽1, \end{array}\right.$$

*p*_∗*k*_ is the myocardium pressure, *A*_0__*k*_ is the unstressed cross‐sectional area, and *c*_*k*_ is the parameter of the vessel wall elasticity.

At the aortic root the blood flow is defined as a function of time, approximating the data from^71^

(11) $$u(t,0)A(t,0)=Q_{H}(t).$$

It corresponds to the heart rate of 1 Hz and stroke volume of 60 ml. We scale the period of this function according to the measured heart rate, and adjust the amplitude $\left({\bar{Q}}_{H}=\max Q_{H}(t)\right)$ by fitting measured and simulated systolic and diastolic blood pressure.

At vessel junctions, the continuity of total pressure is utilised

(12) $$p_{i}\left(A_{i}\left(t,{\tilde{x}}_{i}\right)\right)+\frac{\rho u_{i}^{2}\left(t,{\tilde{x}}_{i}\right)}{2}=p_{j}\left(A_{j}\left(t,{\tilde{x}}_{j}\right)\right)+\frac{\rho u_{j}^{2}\left(t,{\tilde{x}}_{j}\right)}{2},$$

together with the required compatibility conditions along the outgoing characteristics for equations 7 and 8, where *I*,*j* are the indices of the vessels at the junction, and $\bar{x}$ is the coordinate of the terminal point.

Each terminal artery with an index *k* is connected to the venous pressure *p*_*veins*_=12 mmHg through the hydraulic resistance *R*_*k*_

(13) $$p_{k}\left(A_{k}\left(t,{\tilde{x}}_{k}\right)\right)-p_{veins}=R_{k}A_{k}\left(t,{\tilde{x}}_{k}\right)u_{k}\left(t,{\tilde{x}}_{k}\right).$$

Parameters *R*_*k*_ are adjusted to reproduce the arterio‐venous pressure drop.

A stenosis is included as a separate vessel with a smaller diameter *d*_*sten*_, dependent on the degree of the stenosis *α* from the patient‐specific geometry

(14) $$d_{sten}=d_{non-sten}(1-\alpha ),$$

where *d*_*non*−*sten*_ is the diameter of the neighbouring normal vessel. No additional pressure drop model for the stenosis is introduced.

Compression of some coronary arteries during systole by myocardium is an important feature of the coronary haemodynamics. To account the compression, we set in (9) $p_{*}=P_{ext}^{cor}(t)$. We assume that the dependence $P_{ext}^{cor}(t)$ is similar to the heart outflow time‐profile (11). It is scaled so that the maximum value $p_{*}^{max}$ corresponds to the ventricular pressure. For the terminal vessels of the LCA and the RCA, we set $p_{*}^{max}=120\;\text{mmHg}$ and $p_{*}^{max}=30\;\text{mmHg}$, respectively. The terminal resistance *R*_*k*_ in (13) during systole is set to $R_{k}^{syst}=3R_{k}^{diast}$, where $R_{k}^{diast}$ is the terminal resistance during diastole.^72^

We simulate the hyperaemic conditions by performing a 20% reduction of *c*_*k*_ in (9) and halving *R*_*k*_ in (13). This provides a 3‐ to 4‐fold increase of the coronary blood flow which corresponds to typical clinical observations.^44^

## 3 RESULTS

This section begins by reporting the results of each framework separately. However, due to the small number of patients/lesions in the study, it is not feasible to investigate diagnostic performance for each method, nor is this the goal of the present work. Hence, an additional section studying the overall diagnostic performance will be given.

The analysis involves the mean absolute difference (MAD), calculated as

(15) $$\text{MAD}=\frac{1}{n}\sum_{i=1}^{n}\left|{\text{FFR}}_{i}-{\text{cFFR}}_{i}\right|,$$

where cFFR is the model prediction, FFR is the invasive clinical measure, and *n* is the number of lesions assessed. The mean absolute difference, mean difference, and Pearson correlation coefficient are shown in Table 4. The correlation plots and Bland‐Altman (difference plots) are also given for each framework. The diagnostic performance is studied for the pooled results of all workflows. Table 5 compares cFFR of all methodologies to the measured FFR for all lesions, while Figure 7 visualises the FFR results from the table. The Pearson correlation coefficients between all computational workflows are presented in Table 6. The percentage of blood flow estimated by each workflow distributed from the LCA to the LAD and LCx is shown in Table 7.

**Table 4:** Performance overview of cFFR methodologies 1 to 4 compared with the invasive FFR measurements

	Method 1	Method 2	Method 3	Method 4
Mean difference	−0.0179	−0.0129	0.0016	0.2900
Mean absolute difference	0.0650	0.1057	0.0984	0.2900
Pearson coefficient	0.35	0.38	0.11	0.19

Abbreviation: FFR, fractional flow reserve.

**Table 5:** Comparison of clinically measured FFR with cFFR methodologies 1 to 4

Patient no.	Lesion no.	Lesion Location	FFR	Method 1	Method 2	Method 3	Method 4
1	1	LAD	0.89	0.75	0.7	0.71	0.56
2	2	LAD	0.86	0.93	0.75	0.89	0.53
3	3	RCA	0.88	0.81	0.95	0.85	0.23
4	4	LAD	0.82	0.85	0.59	0.84	0.26
5	5	LAD	0.82	0.85	0.77	0.71	0.59
6	6	LAD Prox	0.90	0.96	0.97	0.97	0.85
6	7	LAD Dist	0.82	0.86	0.91	0.90	0.39
6	8	DA	0.81	0.75	0.78	0.59	0.37
7	9	LAD	0.75	0.86	0.91	0.92	0.63
7	10	LCx	0.84	0.78	0.97	0.68	0.53
8	11	LAD	0.88	0.95	0.96	0.88	0.72
8	12	LCx	0.89	0.92	1.00	0.9878	0.79
9	13	LAD	0.83	0.89	0.95	0.89	0.6
10	14	LAD	0.72	0.8	0.68	0.87	0.6

Abbreviations: DA, diagonal artery; FFR, fractional flow reserve; LAD, left anterior descending artery; LCx, left circumflex artery; RCA, right coronary artery.

**Table 6:** Pearsons linear correlation coefficient between all computed workflows

	Method 1	Method 2	Method 3	Method 4
Method 1	1	0.41	0.78	0.57
Method 2		1	0.37	0.42
Method 3			1	0.43
Method 4				1

*Note*. Only the top right values are given as there is diagonal symmetry

**Table 7:** Flow distribution to the LAD and LCx for each methodology

	Blood flow distribution through left coronary arterial network (*%*)							
	Method 1		Method 2		Method 3		Method 4	
Patient number	LAD	LCx	LAD	LCx	LAD	LCx	LAD	LCx
1	44.3	55.7	57.0	43.0	46.0	54.0	24.1	75.9
2	53.4	46.6	68.0	32.0	58.3	41.7	94.4	5.6
3	−	−	−	−	−	−	−	−
4	25.8	52.4	78.6	21.4	54.1	45.9	27.6	51.7
5	36.3	63.7	66.5	33.5	50.2	49.8	56.25	43.75
6	53.2	46.8	70.3	29.7	55.2	44.8	37.5	62.5
7	40.6	59.4	70.1	29.9	59.9	40.1	46.4	53.6
8	71.7	28.3	86.8	13.2	42.9	57.1	65.9	34.1
9	45.2	54.8	67.5	32.5	71.5	28.5	35.1	64.9
10	41.7	58.3	57.1	42.9	53.4	46.6	42.9	57.1

*Note*. Patient 3 is not considered as the lesion is located in the RCA.

Abbreviations: LAD, left anterior descending artery; LCx, left circumflex artery; RCA, right coronary artery.

### 3.1 Comparison of methodologies

As stated in Section 2.1, several aspects involved in the computation of cFFR were left open for each methodology to define. A comparison of such is presented here.

In order to compare boundary conditions, flow rates within the major coronary arteries are considered for each methodology in Table A1. The locations of the flow rates considered are, for a stenosis in the left side of the coronary network, the left coronary artery, the left anterior descending artery, and the circumflex artery; while for a stenosis in the right side of the coronary network, the right coronary artery. In addition, the mean flow rate distal to the lesion is also considered, which allows a comparison of resistance distribution within the coronary network.

#### 3.1.1 Coronary haemodynamics

In this section, we analyse the flow rates predicted by the models, and together with the model‐predicted FFR values, they are used to compare the various boundary conditions used for each methodology. In general the predicted mean flow rates for methodology two (3D model) were significantly larger in magnitude than the other methodologies (reduced‐order 1D/0D models).

For patient 1, all methodologies overpredicted the impact of the stenosis on pressure when compared with the clinically measured FFR. All methodologies predicted a positive FFR (less than 0.8), while the clinical measurement was a significantly higher value of 0.89. In this case, there are actually multiple stenoses within the LAD, with a stenosis located both before and after the origin of the first diagonal artery, while the FFR measurement is taken much further down the LAD after the second diagonal artery. The model predicted mean flow rates at the location of cFFR vary significantly among the methodologies, with the method 1 estimate being 0.79 ml/s, method 2 estimated 3.02 ml/s, method 3 estimated 1.8 ml/s, and method 4 estimated 0.5 ml/s.

Comparing with results for patient 2, methodologies 1 and 3 correctly predicted a negative FFR value (greater than 0.8), while methods 2 and 3 incorrectly predicted a positive FFR value. Patient 2 had a significant blockage in the LCx (no FFR measurement performed), and a moderate narrowing in the LAD, for which FFR was measured. The flow rates in the LCA are again significantly larger for methodology two, when compared with the other method predictions.

Patient 3 had three stenoses at locations throughout the RCA, with a single clinical FFR measurement taken distal to the last stenosis. Methodologies 1 to 3 correctly predicted a negative FFR value. The inlet mean flow rates varied for each methodology from 0.51 ml/s in method 4 to 4.53 ml/s in method 2. The model predicted flow rates at the location of the cFFR were similar for methodologies 1 and 2, while the remaining methodologies predicted a lower mean flow are at this location.

Patient 4 has a single long stenosis located in the LAD. Methodologies 1 and 3 correctly predicted the negative FFR value. The sum of the flow rates in the LAD and LCx for methodologies 1 and 4 do not equal the mean flow rate in the LCA as these two workflows considered the junction to have another artery, and hence three child vessels (LAD, LCx, and intermediate artery) were considered at the junction in these two workflows.

The stenosis of patient 5 is located in the LAD at the junction with the first diagonal artery. Only methodology 1 correctly predicted a negative FFR value under blind‐testing conditions. Compared with workflows 2 and 3, methodology 1 estimated a significantly lower flow that was distributed to the LAD from the left coronary artery, which resulted in a lower flow rate prediction in the LAD and a higher FFR value.

For patient 6, the clinical FFR measurements were performed at three locations. All methodologies correctly predicted a negative FFR value for the proximal LAD; methodologies 1 to 3 correctly predicted the negative FFR of the distal LAD; while none of the methods could correctly predict the positive FFR value in the diagonal artery where the lesion was located very close to the vessel junction with the LAD; although the FFR prediction for workflow 2 was only 0.03 from the invasive measurement.

Two FFR measurements were taken for patient 7. Only methodology 4 correctly predicted a positive FFR value for the stenosis located in the LAD. While only methodology 2 correctly predicted a negative FFR value for the LCx. Interestingly, method 2 (3D model) was the only methodology to correctly predict that the FFR value in the LCx is greater than the FFR value in the LAD.

Patient 8 also had two clinical FFR measurements taken. One in the LAD and the other in the LCx. Methodologies 1 to 3 all correctly predicted a negative FFR value for both stenoses. The flow rate predicted at the inlet of the LAD for methodology 3 was significantly lower than workflows 1 and 2; yet workflow 3 correctly predicted the FFR value to be 0.88, while workflows 1 and 2 predicted much higher FFR values.

Patient 9 has a stenosis located in the LAD. Methodologies 1 to 3 correctly predicted a negative FFR value. Methodology 3 estimated the highest flow rates at the inlet of the LAD and at the location of cFFR, yet predicted the same FFR value as workflow 1. The estimated flow distributions from the left coronary artery to the LAD and LCx are similar for workflows 2 and 3.

Patient 10 also has an FFR measurement taken in the LAD. Methodologies 2 and 4 correctly predicted a positive FFR value. Methodology 2 estimated significantly higher flow rates at the inlet of the LAD than the other workflows, although the distribution of flow to the LAD and LCx from left coronary artery was again quite similar between workflows two and three.

### 3.2 Flow rate and FFR

It would be expected that for a given vessel geometry, an increased flow rate across a stenosis would result in a lower FFR value. Thus, we test the hypothesis that the methodology which predicts the largest flow rate across a stenosis would predict the lowest FFR value. This hypothesis only holds for two FFR cases (patients 1 and 5), while fails for the remaining 12 lesions. An interesting situation occurs for FFR prediction of patient 6 for the lesion located in the proximal LAD. The estimated flow rates are significantly different for modelling approaches 1 to 3, but their predicted FFR values are very similar.

In general, the hypothesis that the greater the flow rate across the stenosis, the more negative the predicted FFR, fails. This indicates that many other factors are involved when estimating cFFR through modelling approaches. These factors include (a) the quality of segmentation and (b) assumptions of the model such as dimensionality, compliance, and resistance estimations for the vessels within the coronary network, and estimation of boundary conditions.

### 3.3 Overview of results

#### 3.3.1 Methodology 1

**Figure 3:**
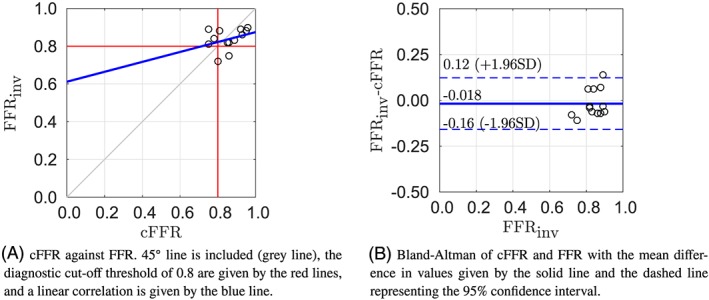
Results of methodology 1

Figure 3A compares the cFFR values with the measured FFR. Approach 1 has a Pearson product‐moment of 0.35, which shows a moderate positive correlation. This pipeline showed the lowest mean absolute difference between cFFR and measured FFR. However, the framework failed to predict the positive FFR value (measured FFR<0.8) for patients 7 and 10. The figure is separated into quadrants via the red line, with the bottom left corresponding with true positive FFR predictions, bottom right corresponding to false positives, top left corresponding with false negatives, and top right corresponding to true negative. The Bland‐Altman plot shown in Figure 3B also features the 95*%* confidence interval of the mean difference. Methodology 1 has the narrowest limits of agreement of all the methods. The cFFR estimation of patient 1 lies outside the confidence interval. This methodology has a negative bias which indicates that the cFFR is generally slightly overestimated, while the severity of the stenosis is underestimated.

#### 3.3.2 Methodology 2

**Figure 4:**
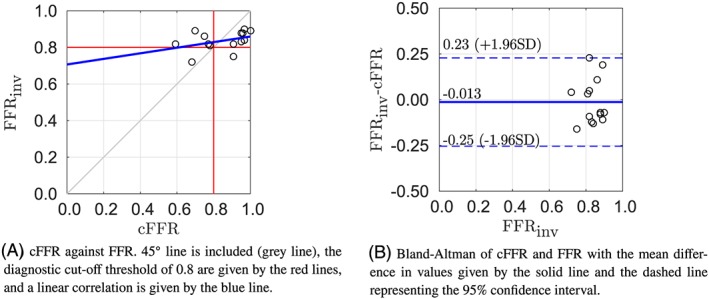
Results of methodology 2

The Pearson product‐moment for this framework is 0.38, which indicates a moderate positive correlation. Figure 4A compares the cFFR values of this approach with the measured FFR. This method correctly predicted one of the cases for which the FFR was under to diagnostic cut‐off point of 0.8. Figure 4B shows the Bland‐Altman plot with a 95*%* confidence interval of the mean difference. The limits of agreement are quite narrow, while the cFFR of patient 4 lies outside of this confidence interval. The method has a negative bias which indicates that the cFFR is generally slightly overestimated, while the lesion severity is underestimated.

#### 3.3.3 Methodology 3

**Figure 5:**
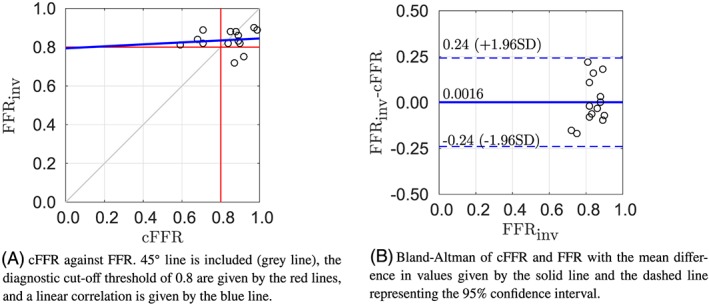
Results of methodology 3

Figure 5A shows the comparison of the cFFR values with the measured FFR. A Pearson product‐moment of 0.11 is found for this framework, which indicates a low positive correlation. The Bland‐Altman plot with a 95*%* confidence interval of the mean difference is shown in Figure 5B. Methodology 3 shows the smallest bias, and the second smallest range for the limits of agreement. The small positive bias indicates that the method generally slightly underestimates the cFFR and thus slightly overestimates the severity of the lesion.

#### 3.3.4 Methodology 4

**Figure 6:**
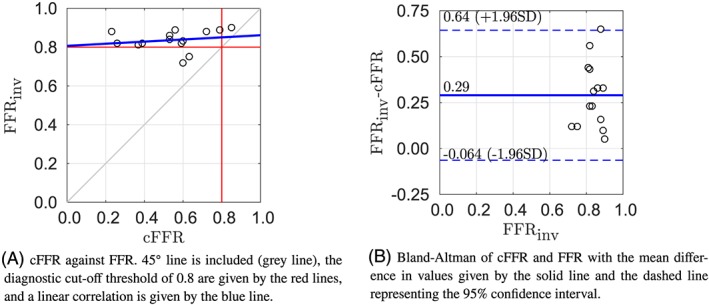
Results of methodology 4

Figure 6A compares the cFFR values with the measured FFR. This method has a Pearson product‐moment correlation of 0.19 which indicates a low positive correlation. The cFFR value is underestimated for all patient cases tested, while the methodology has the largest mean absolute difference. Figure 6B shows a Bland‐Altman plot with a 95*%* confidence interval of the mean difference. Methodology 4 shows the widest limits of agreement, and the positive bias indicates that the method underestimates the cFFR value and overestimates the severity of the stenosis.

A corrected methodology for this framework is described in Appendix B and shows excellent improvement, albeit the corrected framework was developed unblinded with respect to the invasive measurement.

### 3.4 Pooled results

**Figure 7:**
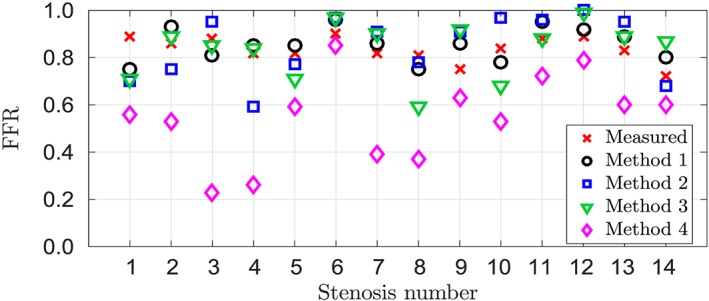
Comparison of measured invasive fractional flow reserve (FFR) with all computational workflows for each stenosis from Table 5

Due to the small number of patients, with a total of 14 lesions, the diagnostic accuracy for each approach was deemed to be uninformative. Thus, for diagnostic performance, the pooled results of all methodological workflows are used, which provides a total of 56 cFFR values. Table 8 shows the diagnostic performance of the pooled results. The positive predictive value was low at 11.54*%*; however, the negative predictive value was at a respectable 83.33*%*. The study showed an overall sensitivity of 37.5*%* and a specificity of 52.08*%*. Due to the low prevalence of positive FFR (14.3*%*), the negative predictive value is easier to match, while matching the positive predictive value is challenging. Figure 7 compares all cFFR predictions of all computational workflows with the invasive FFR measurement.

**Table 8:** Pooled diagnostic results of cFFR showing the total number of true positive, false positive, true negative, sensitivity, specificity, positive predictive value, negative predictive value, positive likelihood ratio, and negative likelihood ratio

Number of true positive	3
Number of false positive	23
Number of true negative	25
Number of false negative	5
Sensitivity, *%*	37.50
Specificity, *%*	52.08
PPV, *%*	11.54
NPV, *%*	83.33
LR positive	0.7826
LR negative	1.2000

## 4 DISCUSSION

Four independent approaches for determining cFFR have been described and compared. These approaches vary from image processing to the definition of boundary conditions and model solution. This has enabled us to perform an objective assessment of the workflows and to investigate the effect of boundary conditions and geometrical modelling assumptions in the cFFR value.

The Bland‐Altman plots indicate that methodologies 1 to 3 gave relatively consistent errors as the limits of agreement were relatively narrow for each of these approaches. However, significant differences were seen in the cFFR values between approaches, which indicates the importance to accurately define a strategy to estimate boundary conditions.

### 4.1 Comparison of methodological procedures and assumptions

Methodologies 1, 3, and 4 are based on reduced‐order modelling, and thus these models can be compared more easily, while methodology two is based on rigid‐wall 3D modelling. In this sense, there have been some recent efforts to compare the predictive capability of reduced order models and 3D models.^23^, ^73^ All the reduced‐order models use transient simulations, while methodology 2 uses steady‐state simulations.

There are several different modelling assumptions in the reduced‐order approaches. Comparing these methodologies, the momentum correction factor which multiplies the term $\partial \left(Q^{2}/A\right)/\partial x$ in the conservation of linear momentum equation for methodologies 1 and 4 is equal to 1, which equates to an assumption of a flat velocity profile, while for methodology 3, it is 4/3, which represents an assumption of a parabolic profile. The viscous friction coefficient for methodology 1 is 22, which assumes a mostly flat velocity profile with a small boundary layer, while methodologies 3 and 4 assume a viscous friction coefficient of 8, which corresponds to a parabolic profile.

The 3D model (approach 2) naturally accounts for the bifurcations in the network, and in turn, reduced‐order approaches require coupling conditions between vessels. Methodology 1 is the only reduced‐order approach which implemented conservation of mass and of static pressure at junctions. Methodologies 3 and 4 impose conservation of mass and conservation of total pressure. The choice of using static pressure or total pressure conservation is still open to debate in the field, with groups using different approaches including adding pressure loss terms. This led to the development of a unified method^74^ that investigated the impact of vessel diameter ratios, flow rates, and the angle between vessels at bifurcations. The study indicated that total pressure was more appropriate for straighter branches (small angle between parent and child vessel), while static pressure was more representative for side branches with larger angles between the child vessel and the parent. Although these tests and the unified method proposed was only implemented for steady‐flow conditions and regular, uniform, smooth geometries, the unified approach (or a modified version of it) could potentially improve estimates of pressure drops at vessel junctions for reduced‐order models, especially if stenoses are present.

It was observed that methodologies 1 and 3 generally had the closest agreement and strongest linear correlation coefficient with the majority of cFFR estimates being within 0.07 for 10 of the 14 lesions. Interestingly, the four outlier cases (stenosis 5, 8, 10) have a stenosis that occurs at the beginning of the vessel near a vessel junction, which indicates that the cFFR estimation may be more sensitive to the estimated pressure conservation law at vessel junctions than of the assumed velocity profile (non‐linear correction term and friction term). The choice of the pressure condition at a junction could become more important if the stenosis is located at the vessel junction, and this is supported here as the largest deviations of methodology one and three occur in cases where the stenosis begins immediately at the start of a child vessel at a vessel junction.

Another variation between the reduced‐order methodologies involves the addition of a lumped pressure‐drop model to better represent additional energy losses as a result of recirculation and flow separation distal to the stenosis. This model was originally constructed from experimental measurements^67^ and has the disadvantage of requiring the stenosis segment to be manually isolated and the pressure‐drop model to be inserted. The reduced‐order modelling approach 3 has included a pressure‐drop model, while methodologies 1 and 4 did not include an additional pressure‐drop model, instead relying on the geometric representation of the stenosis and the standard 1D haemodynamic equations.

None of the methodologies used the same constitutive laws. Approaches 1 and 3 used different visco‐elastic constitutive laws, approach 2 (3D model) assumed rigid wall conditions, while approach 4 assumes a non‐linear elastic relationship which changes depending on whether the area is above or below a given reference area. Coronary arteries experience external pressures during heart muscle contraction, particularly in the intra‐myocardial vessels; in addition, the position of the coronary arteries will vary as depending on the heart contraction state. In this paper, none of the methodologies considered the movement of coronary arteries. However, approaches 1, 3, and 4 did consider external pressures acting on vessels. In approaches 1 and 3, the external pressures are experienced in the complex lumped models, which includes volume‐dependent resistances and variable external pressures, which depend on the left and right ventricular pressure in the lumped parameter heart model. Approach 4 adds external pressure in a selection of 1D vessels, and depends on the heart outflow time‐profile, scaled to match ventricular pressure. Approach 2, which is a rigid‐wall 3D model, does not include any external pressures on the coronary vessels.

Inlet boundary conditions are extremely important to estimate cFFR accurately, which will depend on both coronary pressures and the inlet flow rate. One of the difficulties with using only a single‐phase of CCTA, involves the inability to accurately estimate stroke volume, which would require time‐varying data. Approaches 1 and 3 use closed‐loop reduced‐order modelling to estimate inflow rates to the coronary arteries and pressures in the left and right ventricles. Approach 2 imposes a Neumann boundary condition at the inlet and estimates the total coronary flow rate using clinical data and a formula reported for a general population, while approach 4 defines an inlet flow rate.

The outlet boundary conditions for all methodologies are based on lumped models. For approaches 1 and 3, the 1D terminal vessels are coupled to complex lumped parameter models, which vary resistances in time and experience external pressures from the left and right ventricles. Approaches 2 and 4 are coupled with a resistance lumped model.

### 4.2 Influence of boundary conditions

Other than the quality of the segmentation and therefore accuracy of the geometry (which was the same input data for all models), the inlet and outlet boundary conditions, as well as the flow under hyperemia, are perhaps the most important modelling assumptions.^75^ Unfortunately, there will always be uncertainty in estimating boundary conditions, as even the measurement hardware has significant sources of uncertainty (often ±5−10*%*). The inlet boundary condition can be part‐personalised, as time‐varying imaging techniques can be used to estimate stroke volume, and together with heart rate, the cardiac output can be calculated. However, in this paper, a single‐phase of CCTA was available, and hence there is significant uncertainty with regards to the inflow boundary conditions each methodology estimates. In general, methodology 2 defines a larger inlet flow rate in the LCA when compared with the other approaches; this is not the case for patient 9, where methodology 3 imposes a larger flow rate. Due to similar mean differences of cFFR to clinically measured FFR between methodologies 1 and 2, while the flow rates in approach 1 is often close to half the value of approach 2, it can be concluded that based on the pressure drop, the first approach estimates a larger resistance of such major vessels, when compared with the pressure drop from the 3D simulation in approach 2.

The distribution of resistance throughout the network is also an important aspect for accurately estimating the FFR as it effects the flow distribution in the coronary network and determines the flow rate through the interrogated vessel. Methodologies 1 and 2 use similar estimations of the distribution of resistance to terminal vessels, using Murray's law with exponents 2.27 and 2.66, respectively. Both use the radius at the proximal end of the artery; however, approach 2 uses the ostium (the point at which vessel originates), while approach 1 uses a point approximately 0.5 cm into the vessel (the proximal part of these vessels are often in the shape of a funnel). For some patient cases, this has caused significant differences in the flow distribution from the LCA to the LCx and LAD. For example, for patient 1, the flow percentage to the LAD and LCx is 44.3*%* and 55.7*%*, respectively, for approach 1, while for approach 2, it is 57.0*%* and 43*%*, respectively. The difference is even more extreme for patient 7, where the flow percentage to the LAD for approach one is 40.6*%* and is 70.1*%* for approach 2. This observation underlines the importance of developing accurate and appropriate estimations of blood flow distribution within the coronary network, as these two slightly different estimation techniques produce significantly different blood flow distributions.

### 4.3 Limitations

The main limitations of this study are the size of the patient/lesion sample (*n*={10,14}) and the low prevalence of positive FFR measurements (*n*=2). In this context, statistical and diagnostic indexes are sensitive to few changes in results.

### 4.4 Data availability

All available data utilised by the four research groups involved in this study, plus the invasive measurements of FFR, are freely available for download at https://doi.org/10.6084/m9.figshare.8047742.v2. The data includes 3D surface meshes, patient clinical data with FFR and cFFR values (Table 1 and Table 5), and sketches from physicians indicating the approximate location of FFR measurement.

## 5 CONCLUSIONS

This benchmark study of FFR is the first of its kind and attempts to provide a comparison concerning the computation of FFR through mathematical models, which is homogeneous in the sense of the clinical data employed for the construction of the models. The data required to implement each case, including the geometric models of coronary networks, patient clinical data, and measured and computed FFR values, are supplied in supplementary information for this study and is anticipated to be an important and useful resource to speed‐up research by other groups.

A comparison of four different non‐invasive cFFR frameworks was investigated. Significant differences were seen between results of these frameworks, showing the importance of modelling assumptions which are made within each framework. Approach 1, which uses a 1D‐0D modelling methodology, showed the least overall deviation of cFFR values from the clinical measurements, although the results of methodologies 2 (3D model) and 3 (1D‐0D model) were of a similar accuracy. The results indicate that reduced‐order 1D‐0D modelling could be used effectively to estimate FFR values. The addition of a pressure‐drop model for cFFR in reduced‐order models may not be needed as the accuracy of cFFR was similar whether or not a pressure‐drop model was utilised, while the addition of a lumped pressure‐drop model also requires manual isolation of the stenosis segment in order to add to the model. It was observed that the influence of the vessel junction pressure continuity conditions caused differences between the reduced‐order models in cases where the stenosis was located close to the ostium of a child vessel. In the light of the findings reported in this study, we note that the prediction of FFR using numerical simulation definitely continues to deserve further efforts. The main conclusion we can draw is that the results feature a not‐so‐evident sensitivity with respect to the methodology for defining geometry and boundary conditions. We focused on the comparison of different flow rate conditions as given by the different methodologies. Complementary analyses incorporating the sensitivity to the image processing should be performed to gain insight about the role of the different ingredients in the models.

## Figures and Tables

**Figure B1:**
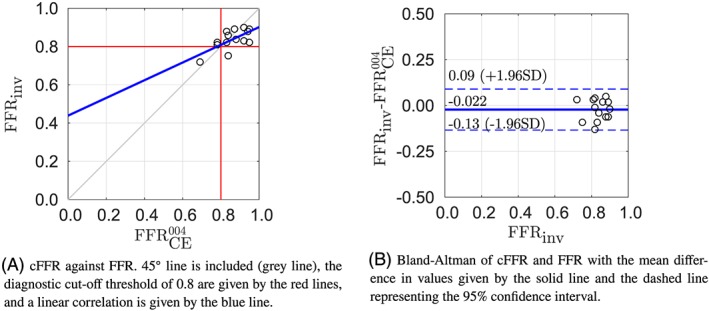
Results of corrected methodology four

## APPENDIX A CORONARY HAEMODYNAMICS

### A.1

**Table A1:** Comparison of the computationally predicted flow rates [mL/s] for each methodology at different locations within each patient specific coronary network

Group	Patient	Stenosis	Inflow LCA	Inflow LAD	Inflow LCx	Inflow RCA	Flow at cFFR Location
1	1	LAD	7.02	3.11	3.91		0.79
2	1	LAD	18.70	10.65	8.05	2.08	3.02
3	1	LAD	5.13	2.36	2.77	3.65	1.80
4	1	LAD	2.90	0.70	2.20		0.50
4c	1	LAD	4.80	1.80	3.00		1.00
1	2	LAD	4.37	2.33	2.04		1.24
2	2	LAD	10.72	7.29	3.43	2.35	2.40
3	2	LAD	6.57	3.83	2.74	3.60	3.30
4	2	LAD	1.80	1.70	0.10		0.50
4c	2	LAD	2.30	2.20	0.10		1.30
1	3	RCA				3.17	1.75
2	3	RCA	19.42	11.06	8.36	4.53	2.05
3	3	RCA	7.16	4.40	2.76	1.87	0.25
4	3	RCA				0.51	0.22
4c	3	RCA				1.80	0.70
1	4	LAD	4.33	1.12	2.27		0.35
2	4	LAD	13.50	10.61	2.89	2.96	3.85
3	4	LAD	5.99	3.24	2.75	3.62	1.89
4	4	LAD	2.90	0.80	1.50		0.30
4c	4	LAD	4.90	1.70	2.10		0.80
1	5	LAD	5.78	2.10	3.68		0.99
2	5	LAD	13.12	8.73	4.39	1.35	1.05
3	5	LAD	5.54	2.78	2.76	3.63	1.78
4	5	LAD	1.60	0.90	0.70		0.30
4c	5	LAD	6.90	3.00	3.90		1.90
1	6	LAD Prox	4.93	2.62	2.31		2.62
	6	LAD Dist					0.88
	6	DA					0.76
2	6	LAD Prox	10.21	7.18	3.03	2.24	7.18
	6	LAD Dist					1.75
	6	DA					2.23
3	6	LAD Prox	6.14	3.39	2.75	3.62	3.25
	6	LAD Dist					2.86
	6	DA					0.25
4	6	LAD Prox	0.80	0.30	0.50		0.30
	6	LAD Dist					0.18
	6	DA					0.12
4c	6	LAD Prox	2.60	1.60	1.00		1.60
	6	LAD Dist					0.85
	6	DA					0.75
1	7	LAD	5.01	2.04	2.97		1.23
	7	LCx					1.81
2	7	LAD	7.33	5.14	2.19	1.61	2.39
	7	LCx					0.51
3	7	LAD	2.37	1.42	0.96	3.72	1.40
	7	LCx					0.50
4	7	LAD	1.40	0.65	0.75		0.40
	7	LCx					0.13
4c	7	LAD	4.80	2.30	2.50		1.60
	7	LCx					0.70
1	8	LAD	6.43	4.61	1.82		0.94
	8	LCx					0.21
2	8	LAD	9.85	8.55	1.30	2.16	1.18
	8	LCx					0.06
3	8	LAD	2.89	1.24	1.65	3.70	0.87
	8	LCx					1.62
4	8	LAD	4.40	2.90	1.60		0.70
	8	LCx					1.00
4c	8	LAD	6.40	4.30	2.10		1.05
	8	LCx					1.10
1	9	LAD	4.66	2.11	2.55		0.78
2	9	LAD	7.51	5.07	2.44	1.65	1.60
3	9	LAD	9.34	6.68	2.66	3.48	2.00
4	9	LAD	3.70	1.30	2.40		0.40
4c	9	LAD	6.70	2.40	4.30		1.45
1	10	LAD	4.04	1.68	2.35		0.69
2	10	LAD	10.17	5.81	4.36	2.23	2.02
3	10	LAD	5.90	3.15	2.75	3.62	2.94
4	10	LAD	1.40	0.60	0.80		0.25
4c	10	LAD	3.70	2.23	1.45		0.70

Abbreviations: DA, diagonal artery; FFR, fractional flow reserve; LAD, left anterior descending artery; LCx, left circumflex artery; RCA, right coronary artery.

## APPENDIX B CORRECTED METHODOLOGIES

### Corrected methodology 4

The only correction within methodology 4 is the new approach to the simulation of hyperaemia. The initial approach was based on the a priori reduction of the peripheral resistance and vascular stiffness: decreasing of *c*_*k*_ in (9) by 20% and decreasing of *R*_*k*_ in (13) by 50%.

The new approach to simulation of hyperaemia is based on the following algorithm:

1. all stenoses are removed,
2. diameters of all coronary vessels are gradually increased until cFFR coefficient becomes greater than 0.95 everywhere in the network without lesions, and
3. stenoses are returned to the model and cFFR is computed.

Thus, we increase the diameters of the vessels instead of decreasing peripheral resistance and vascular stiffness.

### Corrected methodology 4 results

Figure B1A compares the cFFR values with the measured FFR. Methodology 4 has a Pearson product‐moment correlation of 0.66, which indicates a moderate‐strong positive correlation. The mean absolute difference dropped from 0.29 to 0.049. Figure B1B shows a Bland‐Altman plot with a 95*%* confidence interval of the mean difference. The limits of agreement are the narrowest of all methodologies, although this methodology in unblinded, while a positive bias observed where the cFFR is generally overestimated, while the severity of the stenosis is underestimated. A mathematical model of hyperaemia influences drastically the cFFR. Within framework 4 (Figure 6), the cFFR values were computed under the assumption of 3‐ to 4‐fold hyperaemic increase of coronary blood flow. The increase was achieved by the reduction of the peripheral resistance and the rigidity of the vessels. The comparison with the measured FFR values suggested a new approach to hyperaemia simulation: diameters of coronary vessels must be increased until the cFFR values in the network without lesions achieves 0.95 everywhere. The model of framework 4 with the new approach (Figure B1) for simulation of hyperaemia shows excellent improvements, albeit the corrected framework was developed unblinded. The inflation coefficient, defined as the ratio between inflated diameters and initial diameters, has an average value is 1.9, with the lowest value 1.6 for patient 2 and the highest value 2.4 for patient 5 (Table B2). This exceeds the observed physiological inflation during hyperaemia (about 1.15‐1.2), but the methodology is aimed at accurate estimation of the FFR and not specifically at detailed simulations of the hyperaemic coronary flow.

**Table B2:** Inflation coefficient (the ratio between inflated diameters and initial diameters) for all patients

Patient	1	2	3	4	5	6	7	8	9	10
Inflation coefficient	1.7	1.6	1.6	2.3	2.4	2.1	2.0	1.9	1.7	2.1
